# Effectiveness of Preemptive Chlorhexidine Digluconate-Flurbiprofen Spray on Postoperative Sore Throat and Hoarseness in Patients Undergoing Rhinoplasty: A Retrospective Study

**DOI:** 10.7759/cureus.29448

**Published:** 2022-09-22

**Authors:** Fatma F Kartufan, Alpaslan Yildirim, Okan Morkoc, Müslüm Çiçek

**Affiliations:** 1 Anesthesiology and Reanimation, Istinye University Medical Park Gaziosmanpasa Hospital, Istanbul, TUR; 2 Otolaryngology, Finest Private Clinic, Istanbul, TUR; 3 Otolaryngology, Drom Private Clinic, Istanbul, TUR; 4 Anesthesiology, Istinye University, Istanbul, TUR

**Keywords:** hoarseness, sore throat, flurbiprofen, chlorhexidine digluconate, preemptive analgesia, rhinoplasty

## Abstract

Background

In this retrospective study, we aimed to investigate the effect of chlorhexidine digluconate-flurbiprofen spray (Klorhex Plus® oral spray) on postoperative sore throat (POST) and hoarseness (POH) in patients undergoing rhinoplasty.

Methodology

Patients who underwent rhinoplasty alone in our clinic between April 01, 2021, and February 28, 2022, were enrolled in the study. Patients’ demographics such as age, gender, height, and smoking status that could affect sore throat, difficult intubation, and operation time were recorded from the patients’ files. Patients were grouped as those who received Klorhex Plus oral spray before the surgery (Klorhex P group) and those who did not (control group). POST and POH were recorded from the patients’ postoperative surgery files. Postoperative pain evaluation was performed using the Numerical Rating Scale (NRS) at the postoperative first hour (PPL1h), 12th hour (PPL12h), and first day (PPL1d) from the postoperative nursing files.

Results

A total of 354 patients who underwent rhinoplasty alone were included in this study. No significant difference was found between the groups in terms of the demographic data. Statistically, PPL1h, PPL12h, and PPL1d were significantly lower in the Klorhex P group compared to the control group (for all, p < 0.001). POST and POH were also significantly higher in the control group (both, p < 0.001).

Conclusions

The results of this study indicate that Klorhex Plus oral spray is an efficient agent for preemptive analgesia before rhinoplasty. It significantly decreases the postoperative pain level, POST, and POH. However, further comprehensive prospective studies are needed to introduce Klorhex Plus oral spray to rhinology practice.

## Introduction

A large number of patients undergo rhinoplasty for functional and/or cosmetic reasons. Two major complications following rhinoplasty are postoperative sore throat (POST) and postoperative hoarseness (POH). POST is a frequent complication of general anesthesia and has been reported to occur in 30-70% of patients after tracheal intubation [1]. POST following tracheal intubation is thought to result from mucosal erosion caused by the cuff of the tracheal tube. Moreover, the use of nitrous and the duration of surgery are other factors [2]. The insertion of a tracheal tube also affects the voice, and hoarseness (POH) can be seen for several days following rhinoplasty. Hoarseness may have a negative effect on patients’ satisfaction and their daily activities after discharge [3]. The loss of postoperative days of activity and patient discomfort lead to considerable economic and health-related problems.

Postoperative pain is one of the most important complications following these surgeries. Pavlin et al. reported that 60% of patients undergoing ambulatory procedures experience moderate (>3/10) and 20% experience severe (>7/10) pain [4]. Studies have increasingly focused on the use of preemptive analgesia for postoperative complications such as hoarseness and sore throat in several ambulatory procedures, including rhinoplasty [5]. Gulhas et al. found that the administration of 200 mg dexpanthenol prophylactically before endotracheal intubation was effective in the prevention of POST [6].

Many surgical patients have been reported to be over-prescribed opioids for relieving postoperative pain and POST [7,8]. This might be because of the provider’s desire for reducing patient discomfort and increasing satisfaction. However, opioids have numerous side effects, including respiratory depression, hypotension, and postoperative nausea/vomiting (PONV). This has raised the need for seeking alternative efficient multimodal anesthesia techniques to minimize the use of opioids in the postoperative period [9]. Preoperative administration of analgesics, mainly preemptive analgesia, decreases postoperative pain. Preoperative analgesia inhibits central sensitization caused by surgical or inflammatory stimuli and involves perioperative and postoperative periods [10]. Numerous agents have been tried as preemptive anesthetics before plastic procedures [11-14].

In this retrospective study, we investigated the effect of chlorhexidine digluconate-flurbiprofen spray (Klorhex Plus® oral spray) on POST and POH in patients undergoing rhinoplasty for the first time.

## Materials and methods

Study design and patients

The protocol of this retrospective study was approved by the Local Ethics Committee of our university hospital (3/2022.K-17). A short informed consent form was sent to Turkish and foreign patients to screen patient files. In addition, the necessary permissions were obtained from the hospital management for using archived files. The study was performed in line with the relevant ethical principles of the Declaration of Helsinki.

The number of participants required for the study was calculated to be 210 with a t-test, effect size of 0.5, α error probability of 0.05, and power (1-β error probability) of 0.95 using G power 3.1.9.2 statistical software (available at: https://stats.oarc.ucla.edu/other/gpower/). The minimum number of participants was determined as 240 considering the possibility of the difficulty in accessing the data obtained from the volunteers during the study or the fact that some of the participants were excluded.

Patients aged 18-60 years with American Society of Anesthesiologists (ASA) I and II classes, those who did not experience upper respiratory tract infections (URTIs) and/or sore throat and hoarseness, and who did not require any examination and treatment by an otorhinolaryngologist within the last two weeks were included in the study. Patients <18 years old or >60 years old, those with ASA class ≥III, those who underwent surgery other than rhinoplasty, those who experienced URTIs and/or sore throat and hoarseness, and those who required examination and treatment by an otorhinolaryngologist within the last two weeks were excluded from the study.

Patients who underwent rhinoplasty alone in our Ear-Nose-Throat (ENT) and Plastic-Reconstructive Surgery (PRS) clinics between April 01, 2021, and February 28, 2022, were enrolled in the study. Patients’ demographics such as age, gender, height, and smoking status that could affect sore throat, difficult intubation, use of video laryngoscopy, and operation time were recorded.

In our daily practice, the ENT surgery clinic administers Klorhex Plus® oral spray (Drogsan İlaçları Sanayi ve Tic. A.Ş., Ankara, Türkiye) around the cuff of the intubation tubes to prevent POST and POH with the permission of anesthesiologists, while the other PRS clinic does not. Klorhex Plus oral spray application had been recorded in the patients’ surgery-anesthesiology operational files that facilitated our retrospective screening.

Patients who received Klorhex Plus oral spray before the surgery were grouped as the Klorhex P group, and those who did not as the control group. Evaluation of POST and POH were made through postoperative surgery records of the patients’ files. Evaluation of postoperative pain level (PPL) was performed using the Numerical Rating Scale (NRS) at the postoperative first hour (PPL1h), 12th hour (PPL12h), and first day (PPL1d) from the recordings of postoperative nursing files.

The results were compared between the Klorhex P group and the control group. If the relevant data were not found in the file search, the data section was left blank, and if more than 50% of the data could not be retrieved, the relevant volunteer was excluded from the analysis.

Statistical analysis

Data obtained in this study were evaluated using SPSS version 26.0 (IBM Inc., Armonk, NY, USA). The normality of the data was evaluated using the Kolmogorov-Smirnov test. Mann Whitney U test or Student’s t-test was used in the comparison of the continuous variables depending on the skewness, while categorical variables were compared using the chi-square test. Continuous variables were expressed with descriptive statistics (mean ± standard deviation (SD), minimum and maximum values), and categorical variables were presented as frequency (number, percentage). P-values of <0.05 were considered statistically significant.

## Results

A total of 354 patients who underwent rhinoplasty alone in the ENT and the PRS clinics of our hospital were included in this retrospective study. The mean age of the patients was 30.41 ± 8.04 years. Of all patients, 291 (82.20%) were female, and 63 (17.80%) were male. Patients were divided into the following two groups: those who received Klorhex Plus oral spray as preemptive analgesia (Klorhex P group) and those who did not receive preemptive analgesia (control group). Accordingly, 169 (47.74%) patients were in the Klorhex P group, and 185 (52.26%) patients were in the control group. There were 137 female and 32 male patients in the Klorhex P group, while these figures were 154 and 31 in the control group, respectively.

The mean age was 30.31 ± 7.99 years in the Klorhex P group and 30.50 ± 8.05 years in the control group. No statistically significant difference was found between the groups in terms of age (p > 0.05). The mean weight was 66.93 ± 12.81 kg in the Klorhex P group and 67.30 ± 12.90 kg in the control group. No statistically significant difference was found between the groups in terms of weight (p > 0.05). The mean height was 166.67 ± 7.54 cm in the Klorhex P group and 167.34 ± 7.57 cm in the control group, and the difference was not statistically significant. The demographic features of the groups are presented in Table 1.

**Table 1:** Demographic characteristics of the groups. BMI: body mass index

	Overall		Klorhex P		Control		P-value
	Mean	±SD	Mean	±SD	Mean	±SD	
Age (years)	30.41	8.04	30.31	7.99	30.50	8.05	>0.05
Weight (kg)	67.12	12.78	66.93	12.81	67.30	12.90	>0.05
Height (cm)	167.02	7.53	166.67	7.54	67.30	7.57	>0.05
BMI (kg/m^2^)	23.93	3.39	23.96	3.40	23.91	3.44	>0.05
Operation time (minute)	143.83	31.81	144.02	31.92	143.65	32.24	>0.05

**Figure 1:**
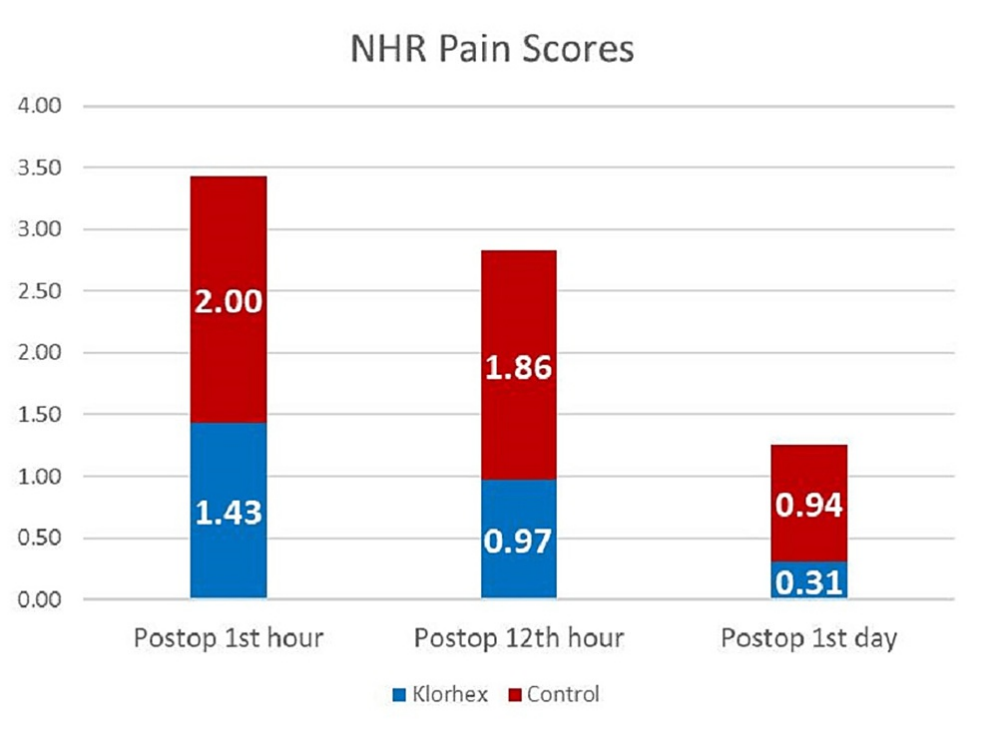
Postoperative pain levels of the groups at different times according to the Numerical Rating Scale.

When the smoking status of the patients was questioned, 28 (16.57%) patients in the Klorhex P group and 59 (31.89%) patients in the control group were smokers. The rate of smokers was statistically significantly higher in the control group (p = 0.001). PPL was measured using the NRS. Accordingly, PPL was statistically significantly lower in the Klorhex P group compared to the control group at PPL1h, PPL12h, and PPL1d (for all, p < 0.001) (Figure 1).

**Figure 2:**
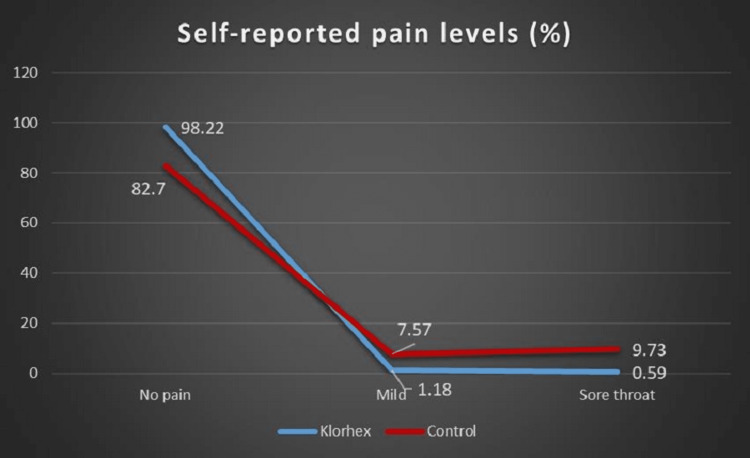
Comparison of postoperative sore throat between the groups.

**Figure 3:**
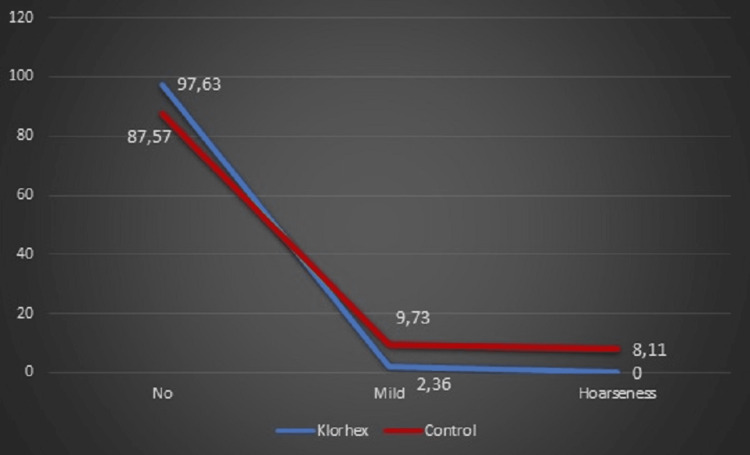
Comparison of postoperative hoarseness between the groups.

The need for non-steroid anti-inflammatory drugs (NSAIDs) was significantly higher in the control group (p < 0.001). POST was evaluated in both groups from recordings and was more common in the control group (p < 0.001). Similarly, POH was evaluated in both groups and was more common in the control group (p < 0.001) (Figures 2, 3).

## Discussion

Postoperative pain management is crucial for ensuring patients’ comfort and satisfaction in any surgery, although this is further important in ambulatory procedures with the expectation to return to normal daily activities as soon as possible. Rhinoplasty is one of the most commonly performed ambulatory surgeries worldwide. It is mainly performed for functional and cosmetic/aesthetic reasons. Rhinoplasty can be performed under various anesthesia methods, including local anesthesia, sedative, or general anesthesia. POST and POH are among the commonly encountered complications following rhinoplasty in general anesthesia and endotracheal intubation. Preemptive analgesia has been proven to decrease postoperative pain [15].

In this study, we evaluated the effect of chlorhexidine digluconate-flurbiprofen spray (Klorhex Plus oral spray) as a preemptive agent. To our knowledge, this is the first investigation of the effects of Klorhex Plus oral spray on POST, POH, and PPL in the literature. According to our findings, Klorhex Plus oral spray provided lower POST and POH compared to the control group.

Various methods have been used to provide preemptive analgesia. Sekhavat et al. used lidocaine infiltration at the incision site and reported decreased postoperative pain levels and the need for opioids postoperatively [16]. Kim et al. investigated the effectiveness of preemptive analgesia with pregabalin in patients undergoing septoplasty and found that pain scores were significantly lower at the first and 12th hours in patients receiving pregabalin tablets. Similarly, in this study, we obtained lower PPL, as measured by NRS, at the postoperative first and 12th hours [17].

In a prospective randomized study, Salama et al. [13] investigated the effect of preemptive gabapentin on anesthetic and analgesic requirements in patients undergoing rhinoplasty. The study revealed that preoperative gabapentin as a single dose of 1.2 g reduced postoperative opioid requirement by 40%. In a meta-analysis, Li et al. reported that intracuff lidocaine and intravenous lidocaine were effective in preventing POST. In addition, intracuff lidocaine was associated with reducing the risk of hoarseness [18]. In a systematic review and network meta-analysis, Yu et al. found that the prophylactic use of nebulized corticosteroids, magnesium, and ketamine can effectively prevent POST [19]. The authors reported decreased postoperative morphine consumption, improved postoperative pain relief, and better patient satisfaction with dexmedetomidine [20].

In this study, Klorhex Plus oral spray significantly reduced the need for NSAIDs compared to the control group; meanwhile, there was no opioid requirement preoperatively or postoperatively in both groups included in the study. On the other hand, the incidence of hoarseness was significantly higher in the control group compared to the Klorhex P group. In total, 23 (12.4%) patients in the control group had hoarseness following rhinoplasty, while this figure was only four (2.37%) in the Klorhex P group. However, we could not compare our findings because of the lack of studies on this issue in the literature.

Study limitations

The major limitations of this study are its retrospective and single-center design. However, the number of patients was relatively high. Finally, we could not perform an exact comparison because no study has investigated the effect of Klorhex Plus oral spray on POST caused by rhinoplasty.

## Conclusions

The results of this study indicate that Klorhex Plus oral spray is an efficient agent for preemptive analgesia before rhinoplasty. It significantly decreases POST and POH. However, further comprehensive studies are needed to introduce Klorhex Plus oral spray to rhinology practice.

## References

[1] Xu YJ, Wang SL, Ren Y, Zhu Y, Tan ZM (2012). A smaller endotracheal tube combined with intravenous lidocaine decreases post-operative sore throat - a randomized controlled trial. Acta Anaesthesiol Scand.

[2] Lee JH, Koo BN, Jeong JJ, Kim HS, Lee JR (2011). Differential effects of lidocaine and remifentanil on response to the tracheal tube during emergence from general anaesthesia. Br J Anaesth.

[3] Yamanaka H, Hayashi Y, Watanabe Y, Uematu H, Mashimo T (2009). Prolonged hoarseness and arytenoid cartilage dislocation after tracheal intubation. Br J Anaesth.

[4] Pavlin DJ, Chen C, Penaloza DA, Buckley FP (2004). A survey of pain and other symptoms that affect the recovery process after discharge from an ambulatory surgery unit. J Clin Anesth.

[5] Arumugam S, Lau CS, Chamberlain RS (2016). Use of preoperative gabapentin significantly reduces postoperative opioid consumption: a meta-analysis. J Pain Res.

[6] Gulhas N, Canpolat H, Cicek M, Yologlu S, Togal T, Durmus M, Ozcan Ersoy M (2007). Dexpanthenol pastille and benzydamine hydrochloride spray for the prevention of post-operative sore throat. Acta Anaesthesiol Scand.

[7] Rose KR, Christie BM, Block LM, Rao VK, Michelotti BF (2019). Opioid prescribing and consumption patterns following outpatient plastic surgery procedures. Plast Reconstr Surg.

[8] Rock AN, Akakpo K, Cheresnick C, Zmistowksi BM, Essig GF Jr, Chio E, Nogan S (2021). Postoperative prescriptions and corresponding opioid consumption after septoplasty or rhinoplasty. Ear Nose Throat J.

[9] Jain PN (2013). Oral pregabalin holds promise to reduce pain after cardiac surgery. Ann Card Anaesth.

[10] Kissin I (2000). Preemptive analgesia. Anesthesiology.

[11] Hall BR, Billue KL, Hon H (2020). No opioids after septorhinoplasty: a multimodal analgesic protocol. Plast Reconstr Surg Glob Open.

[12] Kim D, Jeong H, Kwon J (2019). The effect of benzydamine hydrochloride on preventing postoperative sore throat after total thyroidectomy: a randomized-controlled trial. Can J Anaesth.

[13] Salama ER, Amer AF (2018). The effect of pre-emptive gabapentin on anaesthetic and analgesic requirements in patients undergoing rhinoplasty: a prospective randomised study. Indian J Anaesth.

[14] Chen CY, Kuo CJ, Lee YW, Lam F, Tam KW (2014). Benzydamine hydrochloride on postoperative sore throat: a meta-analysis of randomized controlled trials. Can J Anaesth.

[15] Onal SA, Keleş E, Toprak GC, Demirel I, Alpay HC, Avci L (2006). Preliminary findings for preemptive analgesia with inhaled morphine: efficacy in septoplasty and septorhinoplasty cases. Otolaryngol Head Neck Surg.

[16] Sekhavat L, Behdad S (2011). Preoperative analgesia with local lidocaine for cesarean delivery pain relief. J Matern Fetal Neonatal Med.

[17] Kim JH, Seo MY, Hong SD, Lee J, Chung SK, Kim HY, Dhong HJ (2014). The efficacy of preemptive analgesia with pregabalin in septoplasty. Clin Exp Otorhinolaryngol.

[18] Li H, Yue Y, Qu Y, Mu D (2020). Lidocaine for postoperative sore throat: a meta-analysis of randomized controlled trials. Minerva Anestesiol.

[19] Yu J, Ren L, Min S, Yang Y, Lv F (2020). Nebulized pharmacological agents for preventing postoperative sore throat: a systematic review and network meta-analysis. PLoS One.

[20] Cicek M, Yucel A, Gedik E, Sagir OA, Kadir But AK, Ersoy MO (2006). The effects of intra-operative low-dose dexmedetomidine infusion on postoperative pain in patients undergoing septorhinoplasty. Pain Clin.

